# Should bioprostheses be considered the valve of choice for dialysis-dependent patients?

**DOI:** 10.1186/1749-8090-8-42

**Published:** 2013-03-08

**Authors:** Qiu Zhibing, Chen Xin, Xu Ming, Liu Lele, Jiang YingShuo, Wang LiMing

**Affiliations:** 1 Department of Cardiothoracic and vascular Surgery, Nanjing First Hospital, Nanjing Heart Institute, Nanjing Medical University, 68 Changle Rd, Nanjing, 210006, China

## Abstract

**Background:**

There is controversy regarding the choice of prosthetic valves in patients with cardiac valve disease and dialysis-dependent patients. This review assesses a 12-year experience and outcomes after valve replacement in patients on chronic preoperative renal dialysis, comparing survival and valve-related outcomes following valve replacement with bioprostheses versus mechanical prostheses in this population in china.

**Methods:**

From January 1999 and October 2011, 73 consecutive dialysis patients underwent cardiac valve replacement. The patients were divided into two groups: (Group B) bioprosthesis valves were implanted in 38 (52.1%) patients and (Group M) mechanical valves were implanted in 35 (47.9%) patients. Outcome measures included perioperative data, hospital mortality, major postoperative complications, follow-up outcomes, valve related morbidity and late survival.

**Results:**

There were no significant differences in terms of patient characteristics in the 2 groups. Thirty-three were isolated aortic valve replacements (45.2%); 28 were isolated mitral valve replacements (38.4%); 10 were combined aortic and mitral replacements (13.7%); 2 were combined tricuspid and mitral replacements (2.7%). The overall hospital mortality was 5.5% (n = 4) and was not different between Group B (5.3%) and Group M (5.7%). Low ejection fraction was the only independent predictors of hospital mortality. There was no significant difference between the groups in the overall rate of complications. The overall mean follow-up was 47 ± 23 months. According to the Kaplan-Meier analysis, late mortality, perivalvular leak and freedom from reoperation were similar in patients with mechanical and bioprosthesis valves. The bioprosthesis valve group had significantly higher freedom from thromboembelism-bleeding events (100% versus 77.6 ± 11.0%, p = 0.012), and valve-related morbidity (73.2 ± 10.1% versus 58.1 ± 10.9%, p = 0.035) in 5 years. Kaplan–Meier survival estimates at 1, 3, and 5 years were 0.971, 0.832, and 0.530 in group B, and 0.967, 0.848, and 0.568 in group M.

**Conclusions:**

There is no significant difference in the perioperative morbidity and mortality, late survival of dialysis patients after cardiac valve replacement with bioprostheses versus mechanical valves. In spite of the limited sample size analyzed, its outcome and consistency to several previous reports supports a conclusion that bioprostheses rather than mechanical ones could be a favorable choice for valve replacement needs of renal failure patients.

## Background

Chronic kidney disease population is increasing, so is the dialysis population. Cardiac disease is a major cause of death in patients with end-stage renal disease (ESRD) on hemodialysis [1]. Only 1.5 deaths/1000 patient-years were ascribed to valvular heart disease [2]. Most surgeons believe that mechanical valves are superior to bioprosthetic valves in the setting of chronic renal failure, because of accelerated bioprosthesis calcification and structural degeneration [3,4]. Controversy persists with regard to the optimum choice of prosthesis for valve replacement in dialysis-dependent patients [5,6]. However, the American College of Cardiology/American Heart Association (ACC/AHA) guidelines for valve replacement recommend the use of mechanical valves in dialysis-dependent patients [7].

Given the poor long-term survival of dialysis patients, we reasoned that patients receiving bioprosthese may die before valve failure occur. More recent literature challenges this notion based on the increased risk of stroke and bleeding associated with life-long anticoagulation therapy [8,9]. The purpose of our investigation was, therefore, to analyze our experience with valve replacement in patients on dialysis in order to formulate guideline for choice of valve prostheses.

## Methods

### Patients

The study protocol was approved by the institutional review committee of the Nanjing First Hospital, and informed consent was obtained from all patients. Seventy-three patients requiring chronic hemodialysis underwent valve replacement at Nanjing First Hospital affiliated Nanjing Medical University between January 1999 and October 2011. Patients with acute renal failure, not on chronic hemodialysis, were excluded from this review. Patients who required concomitant coronary artery bypass grafting (CABG) or re-operative surgery were not included in the study.

Mean age was 56.8 ± 14.3 years (range, 28 to 75). Bioprostheses and mechanical prostheses populations were compared through analysis of preoperative variables listed in Table 1.

**Table 1:** Preoperative patient characteristics

**Variable**	**Group B (n = 38)**	**Group M(n = 35)**	***P*-value**
Clinical demographics			
Age (y)	55 ± 8	53 ± 9	NS
Female sex	16 (42.1%)	15(42.9%)	NS
Mean body mass index (kg/m2)	24 ± 5	26 ± 6	NS
Risk factors			
Hypertension	34(89.5%)	32(91.4%)	NS
Diabetes	15(39.5%)	12(34.3%)	NS
Obstructive pulmonary disease	4(10.5%)	3(8.6%)	NS
Previous gastrointestinal bleed	7(18.4%)	6(17.1%)	NS
Peripheral vascular disease	3(7.9%)	2(5.7%)	NS
Mean preoperative creatinine (mg/dl)	4.5 ± 1.2	4.8 ± 1.4	NS
Cardiac profile			
Previous myocardial infarction	2(5.3%)	2(5.7%)	NS
Previous PCI	5(13.2%)	4(11.4%)	NS
Cardiac arrhythmia	27(71.1%)	31(88.6%)	<0.05
Congestive heart failure (NYHA III-IV)	12(31.6%)	11(31.4%)	NS
Mean ejection fraction	0.50 ± 0.12	0.49 ± 0.14	NS
Poor ejection fraction (<0.35)	4(10.5%)	4(11.4%)	NS
Preoperative endocarditis	6(15.8%)	5(14.3%)	NS
Mean EuroSCORE (%)	32 ± 23	31 ± 22	NS

*Group B*, Biprosthesis valve; *Group M*, Mechanical valve; *NS*, not significant.

The decision regarding type of valve placed was made primarily on the basis of the expected survival of the patient. In general, patients who received a mechanical valve were deemed to have a probable survival greater than 5 years. Those with expected survivals less than 5 years were considered primarily bioprosthetic candidates. Other factors such as an inability to tolerate warfarin anticoagulation and individual surgeon’s experience also affected valve selection.

In hemodialysis patients, valve replacement with a mechanical prosthesis is classified as group M, and valve replacement with a bioprosthesis is classified as group B. There were 38 bioprostheses replacements and 35 mechanical prostheses replacements.

### Surgical management

All procedures were performed through a full median sternotomy. Cardiopulmonary bypass(CPB) was established between the ascending aorta and either the right atrium using a two-stage cannula or both venae cavae, and myocardial protection was achieved using high potassium cold blood cardioplegia in an antegrade fashion. During CPB, a perfusion pressure of >60 mmHg and a minimum flow of 2.2 l/min/m^2^ were maintained in all patients. Following surgery, all patients were transferred to the intensive care unit (ICU). Patients were weaned from ventilator when haemodynamic stability was achieved, no postoperative bleeding occurred and adequate consciousness was obtained.

All patients received routine hemodialysis on the day before the operation. Only hemofiltration was conducted during CPB. At the end of CPB, the serum potassium level was < 4.0 mEq · L^-1^. Hemodialysis resumed on the 1st postoperative day. If the patient was hemodynamically unstable, continuous hemofiltration was applied.

### Discharge anticoagulation management

Patients with a mechanical valve were managed with warfarin therapy. Those who had a bioprostheses were also given warfarin for the first 3 months after surgery. Warfarin therapy was started generally on the 2nd postoperative day. No antiplatelet drugs were used for either type of valve. Warfarin was adjusted to maintain an INR of 1.8 to 2.5 after all valve replacements.

### Follow-up

The mean follow-up period was 47 ± 23 months (range, 2–88 months). Late complications were documented wherever possible without bias to the type of valve substitute implanted. Comparison of outcomes between Groups M and B was performed, followed by an analysis of outcome by type and site of valve prosthesis with particular regard to long-term survival.

Specific endpoints are recorded for reporting morbidity and mortality after cardiac valvular operations and include: overall mortality, thromboembolic events, valve thrombosis, structural valve failure, reoperation, prosthetic valve endocarditis (PVE), peravalvular leak and bleeding events. Information obtained from a computer-based valve replacement database, telephone interviews and patient charts was reviewed for follow up data.

### Statistical analysis

Continuous data are presented as the range and, in parentheses, the mean ± standard deviation. Survival estimates were calculated using the Kaplan-Meier method. Actuarial curves were constructed to describe mortality and the incidence of valve-related complications using the Kaplan-Meier technique, and differences between the two groups were compared with the log rank test. A P-value <0.05 was considered statistically significant for all used tests. The statistical analyses were performed with the use of SPSS 15 (SPSS Inc., Chicago, IL, USA). Stepwise multivariate logistic regression was then performed to assess the independent risk factors for hospital mortality and post-operative morbidities.

## Results

### Patient demographics

There were no significant differences between group M and B in terms of patient characteristics, preoperative risk factors, or cardiac profiles (Table 1).

### Intraoperative data

Thirty-five mechanical valves (47.9%) and 38 bioprosthesis valves (52.1%) were implanted. Thirty-three were isolated aortic valve replacements (45.2%); 28 were isolated mitral valve replacements (38.4%); 10 were combined aortic and mitral replacements (13.7%); 2 were combined tricuspid and mitral replacements (2.7%).

There were no differences in terms of CPB and cross-clamp times between the two groups. There was no significant difference in the type of prosthesis implanted when analyzed by dependence on dialysis (Table 2).

**Table 2:** Operative characteristics

**Variable**	**Group B (n = 38)**	**Group M(n = 35)**	***P***-**value**
Cross-clamp time (min)	98 ± 21	101 ± 22	NS
Cardiopulmonary bypass time (min)	132 ± 30	136 ± 33	NS
Operative time (min)	213 ± 42	210 ± 48	NS
Aortic valve replacement	18(47.4%)	15(42.9%)	NS
Mitral valve replacement	15(39.5%)	13(37.1%)	NS
Aortic + mitral valve replacement	4(10.5%)	5(14.3%)	0.035
Tricuspid + mitral valve replacement	1(2.6%)	1(2.9%)	NS

*Group B*, Biprosthesis valve; *Group M*, Mechanical valve; *NS*, not significant.

### Mortality

The overall hospital mortality was 5.5% (n = 4). Hospital mortality was 5.7% (2/35) for mechanical valves, and 5.3% (2/38) for bioprothesis valves. Among the mechanical valve recipients, one died from sepsis and one from pericardial tamponade. Among the bioprothesis valve recipients, one died from mesenteric ischemia and another from hemorrhage during a separate, non-cardiac-related operation.

The univariate predictors of composites of hospital mortality are listed in Table 3. The only significant multivariate independent predictors of hospital mortality was low ejection fraction (<35%) (OR = 3.32, p<0.05).

**Table 3:** Predictors of hospital mortality in multivariate analysis

	**Odds ratio**	**C.I.95%**	**P**-**value**
Ejection fraction < 30%	3.32	1.19–8.95	0.021
Peripheral vascular disease	2.12	1.13–8.22	0.26
Aortic valve replacement	3.18	1.28–9.06	0.13
Prosthesis type	1.57	0.90–3.30	0.08
Age at operation	1.04	1.00-1.06	0.16

*CI*, confidence interval.

### Morbidity

Postoperative complications are shown in Table 4. There was no significant difference between the groups in the overall rate of complications. In-hospital postoperative complications include bleeding requiring exploration in 4; respiratory failure in 4; heart block requiring permanent pacemaker placement in 2; sepsis in 2; myocardial infarction in 2; and pericarditis with effusion and tamponade requiring open pericardial fenestration in 2 patients. The overall rate of reoperation for bleeding was 5.4% and not significantly different between the groups. Patients of Group M, however, presented significantly more often with Gastrointestinal complications (P<0.05). Finally, the median length of ICU stay among patients of Groups B and M was 3.5 ± 1.0 and 3.6 ± 1.2 days, respectively.

**Table 4:** Perioperative mortality and morbidity

**Variable**	**Group B (n = 38)**	**Group M (n = 35)**	***P***-**value**
Hospital mortality (30-day)	2(5.2%)	2(5.7%)	NS
Perioperative myocardial infarction	3(7.9%)	3(8.6%)	NS
Respiratory failure	2(5.2%)	2(5.7%)	NS
Pacemaker placement	1(2.6%)	1(2.9%)	NS
Re-exploration for bleeding	2(5.2%)	2(5.7%)	NS
Cerebral vascular accident	1(2.6%)	1(2.9%)	NS
Sepsis	1(2.6%)	1(2.9%)	NS
Deep sternal wound infection	2(5.4%)	2(5.7%)	NS
Gastrointestinal complications	1(2.6%)	2(5.7%)	<0.05
Length of Intensive care unit stay (days)	3.5 ± 1.0	3.6 ± 1.2	NS

*Group B*, Biprosthesis valve; *Group M*, Mechanical valve; *NS*, not significant.

### Late-term outcomes

**Figure 1:**
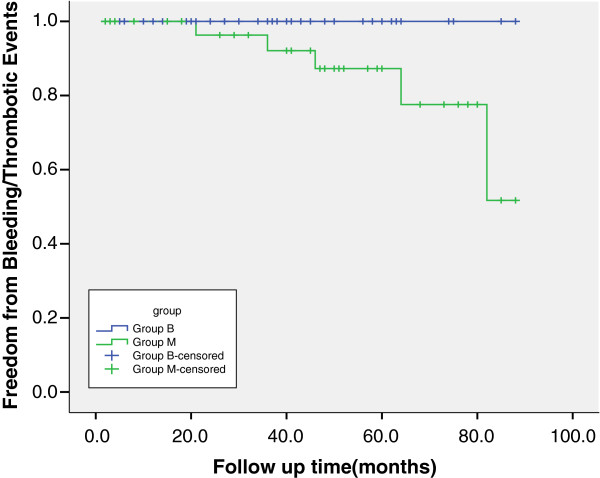
Kaplan-Meier freedom from bleeding/thrombotic events.

**Figure 2:**
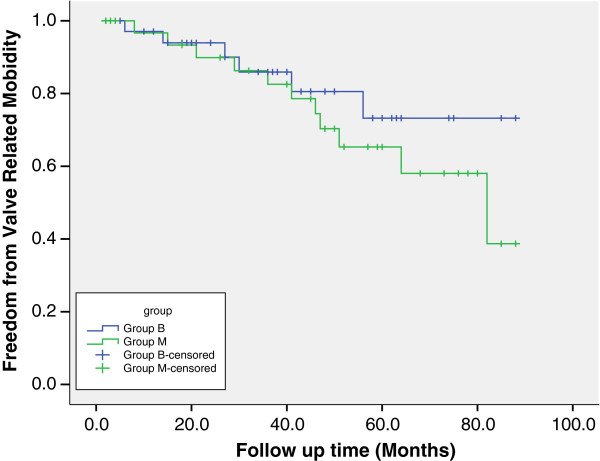
Kaplan-Meier freedom from valve related mobility.

**Figure 3:**
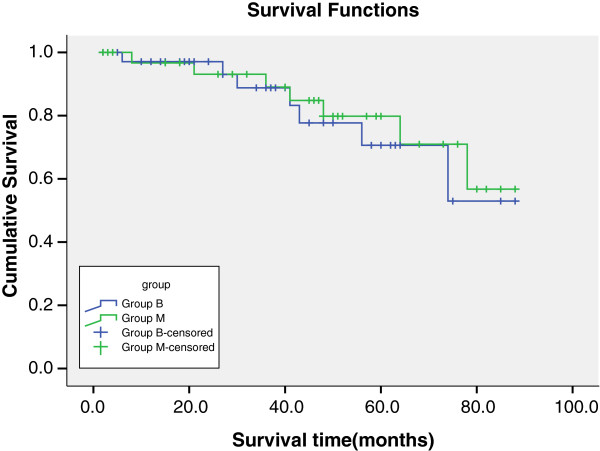
Late-term survival with group B(blue line) versus group M(green line).

Follow-up was completed for all survival patients. The overall mean follow-up was 47 ± 23 months. There were 10 (14.5%) deaths during the total follow-up of 69 patients who survived to hospital discharge, with late mortality of 19.4% in group B and 21.2% in group M. When patients were compared, thrombotic and bleeding events (Figure 1; Table 5), and valve-related morbidity were significantly higher in the mechanical group (Figure 2; Table 5). Kaplan-Meier freedom from mortality (Figure 3; Table 5), PVE (Table 5) and perivalvular leak (Table 5) were similar in both groups. There were no differences in freedom from reoperation between the two groups (Figure 2; Table 5). In the mechanical valve group, two patients underwent reoperation for PVE, and one for perivalvular leak. In the tissue valve group, four patients underwent reoperation, two for PVE, one for perivalvular leak and one for structural valve deterioration. The bioprosthesis valve group had significantly higher freedom from thromboem-bleeding events (100% versus 77.6 ± 11.0%, p = 0.012), and valve-related morbidity (73.2 ± 10.1% versus 58.1 ± 10.9%, p = 0.035) in 5 years.

**Table 5:** Follow-up midterm results

**Complications**	**Group B(n = 36)**	**Group M(n = 33)**	***P *Value**
Valve-related	6(16.7%)	11(33.3%)	<0.05
Bleeding event	0	2(6.1%)	<0.05
Thromboembolism	0	3(9.1%)	<0.05
PVE	2(5.6%)	2(6.1%)	NS
Valve deterioration	0	0	NS
perivalvular leak	1(2.8%)	1(3.0%)	NS
Reoperation	3(8.3%)	3(9.1%)	NS
Cardiac death	2 (5.6%)	2(6.1%)	NS
Heart failure	1(2.8%)	1(3.0%)	NS
Arrhythmia	1(2.8%)	1(3.0%)	NS
Non-cardiac death	5(13.9%)	5(15.2%)	NS
Malignancy	3(8.3%)	2(6.1%)	<0.05
Other	2(5.6%)	3(9.1%)	<0.05
Late mortality	7(19.4%)	7(21.2%)	NS

*Group B*, Biprosthesis valve; *Group M*, Mechanical valve; *NS*, not significant. *PVE*, prosthetic valve endocarditis.

### Survival analysis

In the subset of dialysis patients receiving hemodialysis, there was no difference in survival related to the use of tissue versus mechanical valves. The estimated 5-year survival rate with bioprosthetic valves was 53.0 ± 17.2% versus 56.8 ± 15.5% with mechanical valves. Figure 3 shows Kaplan–Meier survival curves for the entire patient population stratified by study groups. Kaplan–Meier survival estimates at 1, 3, and 5 years were 0.971, 0.832, and 0.530 in group B, and 0.967, 0.848, and 0.568 in group M. However, comparison of survival curves between the two groups revealed no significant difference.

## Discussion

Abnormal calcium homeostasis in patients with end-stage renal failure results in dystrophic calcification. Valvular and perivalvular involvement in ESRD is most commonly manifested as mitral annular calcification and aortic valve calcification [10]. Both mitral and aortic valve calcification occur more frequently and at younger age in those with ESRD than in those with normal renal function [11]. Mitral valve and aortic valve replacement are indicated for severe symptomatic valve stenosis or regurgitation [11].

Valve replacement in hemodialysis patients presents a dilemma to the cardiac surgeon, who must balance the potential for accelerated prosthetic valve deterioration against the morbidity of anticoagulation when selecting a prosthesis [3,5,12]. There is much controversy regarding prosthetic valve selection in patients with ESRD requiring dialysis [6,13,14]. Like other studies [5,9], we found accelerated calcification of bioprosthesis to be uncommon in patients on preoperative dialysis. Importantly, Kaplon et al. found no convincing evidence for accelerated calcification as a major cause of bioprosthetic valve failure and resultant adverse morbidity and mortality [10]. In fact, the limited sample size of this study probably does not prevent it from yielding a meaning conclusion that bioprosthesis could be a preferable choice for ESRD.

Given the lack of a clinical difference in morbidity or survival following either mechanical or bioprosthetic valve replacement, we believe that concern for accelerated calcific bioprosthesis degeneration should not play a role when choosing a valve for patients on dialysis. Rather, other variable such as patient age, gender, level of activity, and presence of infection should dictate valve selection, and not the diagosis of end-stage renal failure. We, however, did not observe differences in preoperative rates of systemic hypertension, smoking history, diabetes mellitus, endocarditis, or cardiac arrhythmias. In this study, therefore, we investigated the risk factors of hospital mortality in dialysis-dependent patients after undergoing cardiac surgery. Univariate analysis showed that preoperative lower LVEF was important independent predictors of hospital mortality. In addition, Kaplan-Meier analysis further accentuated the unacceptably high rates of complications and death with mechanical valves. Recent reports have demonstrated the effective use of tissue valves in dialysis patients, without increased mortality or reoperation compared to mechanical valves [13,15].

Although life expectancy for patients with ESRD has gradually improved in the United States, mortality consistently exceeds 25% per year. Four-year survival of patients on hemodialysis or peritoneal dialysis is approximately 40% [16]. In the present study, the overall survival rate of dialysis patients after isolated valve replacement of 85.2% at 3 years and 55.9% at 5 years is clearly better than in previous reports [17]. This might be due to differences in concomitant procedures and postoperative anticoagulation therapy for those with mechanical valves. Although concomitant CABG was the independent predictors of hospital mortality and survival in the previous reports [5-7,9], we excluded patients with CABG because we tried to clarify the long-term result of isolated valve replacement. Our analysis showed no significant difference in life expectancy or rate of reoperation after follow-up 5 years between patients receiving mechanical or biological prostheses, despite the greater proportion of elderly patients in the biological prosthesis group.

It is clear that the overall survival of ESRD patients was poor. In contrast to previous literature [14,18], differences in survival between patients receiving bioprostheses or mechanical prostheses were related to age at operation and not to prosthesis type. In the present study, we have thus far found no survival difference between bioprostheses and mechanical prostheses patients. And age at operation was found to be no different between the two groups. There was no superiority of freedom from all valve-related complications and individual valve-related complications with mechanical prostheses or bioprostheses. Bioprostheses should not be contraindicated in ESRD patients given the observed rarity of accelerated calcification, as well as the poor intermediate-term survival [18,19].

Chronic dialysis patients tend to have more hemorrhagic complications. Therefore, dialysis patients undergoing anticoagulation therapy may be at increased risk of these complications [5,6]. The target value for anticoagulation therapy is generally lower in China than in Europe and the United States: INR is 1.8–2.5 in the case of dialysis patients in China. This is because minor bleeding complications (such as nasal bleeding or bleeding from cannulation sites) often occur among hemodialysis patients receiving warfarin therapy when their INR exceeds 2.5. During the mean follow-up of 44 ± 26 months, there were bleeding complications in 4 of 20 survivors, and no thromboembolic events occurred. Recently, lower-intensity anticoagulation therapy has been demonstrated to result in a lower rate of bleeding complications with bileaflet mechanical valves, without increasing the rate of thromboembolism [17].

The ACC/AHA guidelines were changed in 2006 to reflect the findings of a series of observational studies which showed no significant difference in the freedom from valve-related events including reoperation and late mortality between patients with dialysis-dependent RF receiving a mechanical or a biological prosthesis [20]. In addition to the above findings, a meta-analysis of the published literature demonstrates no survival difference following valve replacement with either bioprosthesis or mechanical prosthesis in patients with ESRD on dialysis. Some recent studies reported that early structural valve deterioration was uncommon, even in dialysis patients, but others showed that it occurred even in new-generation bioprosthetic valves [21,22]. In fact, these reports also support our current study, which have similar outcomes than the present paper.

### Study limitations

The present study was limited by a lack of randomization, as are all studies of valve replacement in this category of population. Furthermore, as no standardized protocols were used with regard to choice of prosthesis, the data presented herein are subject to individual surgeon biases. In addition, our survival analysis is based on a retrospective study design, and it is possible that selection bias for a particular choice of valve may have occurred.

## Conclusion

In the present study of valve replacement in dialysis patients, tissue valve structural deterioration was negligible, the need for reoperation was similar with either mechanical or tissue valves, and there was no difference in mortality with respect to valve choice. This was most likely because of the very limited life expectancy of a patient beginning dialysis. As a result of these findings, it is recommended that renal failure patients who are on dialysis and require cardiac valve replacement should receive a bioprosthesis.

## Competing interests

The authors declare that they have no competing interests.

## Authors’ contributions

QZB and CX had helped with design of the study, data interpretation and in writing of the paper. XM has made the statistical analysis and took part in the writing process. QZB also took part in the correction of the manuscript according to the reviewers’ suggestions. JYS and WLM had helped in gathering patient information and performed graphic measurements. LLL performed graphics and tables and added comments to the paper. All authors read and approved the final manuscript.
